# Acute Non-Gonococcal Epididymo-Orchitis

**Published:** 1909-07-10

**Authors:** 


### ACUTE NON-GONOCOCCAL EPIDIDYMO-ORCHITIS.

The bacteriology of acute epididymo-orchitis is being elucidated by degrees, but there is still room for much more investigation upon the subject. The commonest cause for the lesion is gonorrhœa, but many cases of acute epididymo-orchitis are due to other causes—mumps or the bacillus typhosus for example.

Time and research lead to the discovery of fresh agents that may attack these parts. Dr. Castellani drew attention to one of these last year, when he described what he calls the "Endemic Funiculitis" of Ceylon. This is a disease in which the whole of the spermatic cord becomes highly inflamed and infiltrated, its circumference increasing to as much as three or even three and a half inches. The tunica vaginalis of the testicle also becomes congested, but usually without leading to acute hydrocele. The cord, when cut across, exudes thick creamy pus both from the pampiniform plexus of veins and from the vas deferens, though the testicle itself generally remains unaffected.

The usual history is that the patient has come home very tired after a hard day's work; he does not feel unwell, and he bathes as usual; after the bath he suddenly begins to shiver, and the shivering develops into a veritable rigor, with a great rise of temperature. Nausea is marked, and there may be actual vomiting; whilst within a very short time pain is complained of along the spermatic cord and in the epididymis. The condition becomes rapidly worse, and the next day or the day after the case is taken to hospital. The general condition looks grave, the temperature is 102° F. or 103° F., and locally there is a large and painful inguinal or inguino-scrotal swelling. Unless care be exerted, strangulated hernia may very easily be diagnosed, especially in those cases in which vomiting and hiccough are marked features. The swelling is hard and very tender. It is non-translucent, and it is dull to percussion. It is usually unilateral.

The penis will naturally be examined critically, but no ulcer or sore is to be found, nor any sign of gonorrhœa. The purulent infiltration of the cord is prone to lead to septicæmia and jaundice unless surgical measures are resorted to, but if the spermatic cord is freely incised as high up as possible the lesion can be cured.

Gonococcal trouble being excluded, it has been the opinion of some that the disease is caused by filaria parasites—a sort of local acute suppurative elephantiasis. Castellani has examined cases from this point of view, however, and he has never succeeded in demonstrating the filarial parasites or their embryos.

On the other hand, this observer examined the pus bacterially in five cases, and in all of them he found the same diplo-streptococcus. That this organism may be the actual cause of the acute epididymo-orchitis in these cases is also suggested by the fact that he examined blood from a vein in two of the five cases, and found the same strain of diplo-streptococcus in the circulating blood as was present in the pus in the spermatic cord.

